# Factors Associated With Rising Homelessness Within US States, 2019 to 2024

**DOI:** 10.1001/jamanetworkopen.2026.5187

**Published:** 2026-04-06

**Authors:** Kathryn M. Leifheit, Leah Robinson, Margaret Nkansah, Peter G. Szilagyi, Craig E. Pollack

**Affiliations:** 1 Department of Health Policy and Management, UCLA Fielding School of Public Health, University of California, Los Angeles; 2 Department of Pediatrics, UCLA David Geffen School of Medicine, University of California, Los Angeles; 3 Department of Health Policy and Management, Johns Hopkins Bloomberg School of Public Health, Baltimore, Maryland; 4 National Clinician Scholar Program, University of California, Los Angeles; 5 Johns Hopkins School of Nursing, Baltimore, Maryland

## Abstract

**Importance:**

Homelessness has risen sharply in the US in recent years, posing a major threat to population health. Although researchers have identified factors contributing to differences in homelessness prevalence between jurisdictions, factors contributing to year-over-year change in homelessness are poorly understood.

**Objective:**

To examine competing explanations for rising levels of homelessness within US states between 2019 and 2024.

**Design, Setting, and Participants:**

This longitudinal cohort study was conducted to measure the association between changing state-level factors and relative change in counts of people experiencing homelessness from 2019 to 2024. The sample comprised adults (aged ≥18 years) and children (aged <18 years) experiencing homelessness in all 50 states and Washington, DC, as measured in the US Department of Housing and Urban Development point-in-time (PIT) homeless count data. Due to PIT count limitations owing to the pandemic, outcome data for 2021 were excluded.

**Exposures:**

State-year average rents, unemployment, pandemic-era housing supports (including emergency rental assistance and eviction moratoria), substance use (proxied by overdoses), immigration, and climate-related property damage were measured via a variety of publicly available datasets.

**Main Outcomes and Measures:**

The primary outcome was year-over-year change in homelessness within states overall, by homelessness type (sheltered or unsheltered), and by age (children or adults).

**Results:**

From 2019 to 2024 (excluding 2021; N = 255 state-years), eviction moratoria and climate-related damages were consistently associated with change in homelessness within the 50 states and Washington, DC. The mean (SD) year-over-year increase in homelessness was 7.0% (22.5%), and there were mean (SD) changes of 6.1% (29.1%) for sheltered homelessness, 16.0% (36.5%) for unsheltered homelessness, 7.2% (20.6%) for adult homelessness, and 8.4% (44.0%) for child homelessness. Each additional 1.0% of person-time covered by a moratorium was associated with a 0.36–percentage point (pp) lower increase in homelessness (95% CI, 0.10-0.61 pp; *P* = .01). Each home-equivalent lost to climate-related events per 10 000 population was associated with a 1.00-pp greater increase in overall homelessness (95% CI, 0.78-1.22 pp; *P* < .001). Results were largely consistent by homelessness type and age. Whereas the mean (SD) year-over-year increase in homelessness was 11.0% (36.4%) for a typical state in 2022, homelessness would have risen by an estimated 19.9% (95% CI, 13.5%-26.3%) absent eviction moratoria, while counts would have risen by only 7.8% (95% CI, 7.1%-8.5%) absent property loss due to climate-related disasters.

**Conclusions and Relevance:**

In this cohort study of 50 states and Washington, DC, measured over 5 years, eviction moratoria and climate-related damages were associated with within-state, relative change in homelessness. These findings suggest that to attenuate surges in homelessness, policymakers should take action to prevent evictions and address climate change.

## Key Points

**Question:**

What state-level factors were associated with rising homelessness in US states from 2019 to 2024?

**Findings:**

In this cohort study including the 50 US states and Washington, DC, each additional 1% of person-time covered by an eviction moratorium was associated with a lower increase in homelessness from 2019 to 2024, and a significant increase was observed for each home-equivalent lost to climate-related events per 10 000 population. By contrast, average rents, unemployment, emergency rental assistance, and substance use were not associated with year-over-year change in homelessness.

**Meaning:**

These findings suggest that to attenuate surges in homelessness, policymakers should take action to prevent evictions and address climate change.

## Teaser

This cohort study investigates whether state-level factors were associated with change in homelessness in the US from 2019 to 2024.

## Introduction

Following a sharp increase in homelessness after the COVID-19 pandemic, a record 1 in 435 people in the US experienced homelessness in 2024.^1^ Surging homelessness carries dire implications for population health, exacerbating chronic physical and mental health concerns,^2,3,4,5,6^ infectious disease risk,^7^ adverse birth outcomes,^8^ and mortality.^9,10,11^ Homelessness also contributes to lower use of preventive care,^2^ increased emergency department visits^12^ and hospitalizations,^13,14^ and higher health care costs.^15^

Rising homelessness has not impacted all populations or US states equally. Whereas overall homelessness rose by 33% nationally between January 2020 and January 2024, people experiencing sheltered homelessness and children experiencing homelessness rose by 40% and 39%, respectively.^16^ State-level change over the same period ranged from an 18% decrease in Wyoming to a 212% increase in Vermont.^16^

Observing these patterns, politicians, reporters, and academics have proposed several explanations for rising homelessness in the US.^17,18^ First, longstanding underinvestment in home construction, together with COVID-19 pandemic–era population mobility, exacerbated the national shortage of affordable housing.^19^ Consequently, rents surged in particular areas while incomes remained stagnant,^20^ placing many at risk for housing insecurity and eviction. Second, unemployment rates, a known driver of homelessness,^21^ spiked early in the pandemic before falling again to low levels.^22^ Third, many argue that increases in homelessness stem from the unwinding of pandemic-era expansions to the social safety net—most directly, the phasing out of housing supports such as emergency rental assistance (ERA) programs, which provided payments to landlords or tenants behind on rent and utilities,^23^ and moratoria blocking evictions.^24^ Fourth, some point to the increasing severity of the substance use epidemic^25^ in the US during this time. Fifth, others assert that rising immigration rates have strained the US housing supply.^26^ Finally, some experts point to increasing climate-related natural disasters, which may contribute to temporary or permanent home loss.^27^

Whereas other studies have identified factors contributing to differences in homelessness prevalence between jurisdictions,^28,29^ our study focuses on year-over-year change in homelessness in the years immediately preceding and following the COVID-19 pandemic. Both prevalence and year-over-year change are key metrics that policymakers, the media, and members of the public use to track homelessness within states. That said, we opted to focus on change, such that our primary question was not, for example, “Why did Hawaii have almost twice California’s prevalence of homelessness in 2024?” but rather, “Why did homeless counts nearly double in Hawaii from 2023 to 2024, while remaining relatively stable in California?” Both questions are important, but the former is better understood: multiple rigorous analyses have demonstrated that economic factors such as housing costs relative to incomes are core drivers of homelessness prevalence within states.^28,29^ By contrast, to our knowledge, factors contributing to change in homelessness have only been explored anecdotally. Drivers of these 2 key metrics may be entirely distinct, with slow-moving structural factors driving prevalence and acute shocks driving year-over-year change.

In this study, we leveraged state-level variation to measure the relative associations of these factors with change in homelessness within states. We conducted analyses for 2019 to 2024, measuring associations between the factors and change in overall homelessness, sheltered homelessness, unsheltered homelessness, and homelessness among adults and children.

## Methods

We conducted this 2-way, fixed-effects cohort study of all 50 states and Washington, DC, using various publicly available data sources to measure change in homelessness among children (aged <18 years) and adults (aged ≥18 years) from 2019 to 2024 and social and structural explanatory variables measured in the preceding years (2018-2023). Per University of California, Los Angeles institutional review board policies, this study was exempt from review due to the use of deidentified data. The study followed the Strengthening the Reporting of Observational Studies in Epidemiology (STROBE) reporting guideline.

### State Year-Over-Year Change in Homeless Counts and Homelessness Prevalence

The US Department of Housing and Urban Development (HUD) requires all jurisdictions to conduct point-in-time (PIT) counts of people experiencing sheltered and unsheltered homelessness to estimate the prevalence of homelessness on a single night in January. HUD provides guidelines for local jurisdictions to estimate the number of people staying in designated shelters and places not ordinarily used as a regular sleeping accommodation.^30^ Although the data are widely considered to undercount homelessness, measurement is typically consistent within states across years and thus appropriate for tracking trends.

We used state-year–level counts to calculate our primary outcome of year-over-year change in homelessness (ie, relative increases or decreases within states between annual counts) (eMethods in Supplement 1). We then measured this same outcome in subgroups by homelessness type (sheltered or unsheltered) and age (children or adults).

We examined 2 secondary outcomes. First, as a relative measure, year-over-year change can produce extreme values in states with low baseline counts of homelessness (ie, small denominators). We therefore examined absolute change in homelessness prevalence as another measure of year-over-year change. Second, measures of annual change are likely to be most sensitive to factors that vary greatly from year to year. These factors likely differ from those associated with overall homelessness prevalence, which we measured as the count per 10 000 population within states.

We measured outcomes over 5 years: from 2019 to 2020 (ie, prepandemic period) and from 2022 to 2024 (postpandemic period). We excluded 2021, since the January 2021 PIT count was limited due to pandemic restrictions.^31^

### Explanatory Variables

We examined state-year variables—all measured in the year immediately preceding PIT counts—as potential factors associated with rising homelessness. These explanatory variables included (1) average rents, (2) unemployment, (3) COVID-19 pandemic–era housing supports (ERA and eviction moratoria), (4) overdose mortality, (5) immigration, and (6) climate events. First, we measured average rents using 2-bedroom fair market rents. HUD calculates these annually, at the county level, based on the 40th percentile of survey-reported gross rents for a typical 2-bedroom apartment.^32^ To derive state-year estimates, we calculated a population-weighted average of county fair market rents.^33^ Second, we measured unemployment as a percentage of a state’s civilian labor force that was unemployed each year (annual averages), as reported by the US Bureau of Labor Statistics.^34^ Third, we measured 2 main forms of pandemic-era housing supports: ERA and eviction moratoria. We measured ERA distribution using quarterly reports from the US Department of the Treasury^35^ and defined coverage as months of rent^33^ provided, relative to the state population of renters earning less than 80% of the area median income.^36^ Eviction moratorium coverage was calculated based on the timing of state^37^ and local^38^ moratoria and the percentage of a state’s population covered by these protections. Moratorium coverage and ERA distribution were coded as zeros for prepandemic years. Fourth, we applied overdose mortality as a marker of the severity of a state’s substance use epidemic, using the Multiple Cause of Death dataset from the Centers for Disease Control and Prevention (CDC) WONDER database for 2018 to 2023.^39^ We measured crude rates of deaths due to drug poisonings (underlying cause of death code X40-X44 or Y10-Y14).^40^ Fifth, we used state-specific American Community Survey data to measure immigration as the count of people who were living abroad 1 year ago per 10 000 population per state.^41^ Finally, we measured climate events as the number of home-equivalents lost to natural disasters per 10 000 population. We calculated this using state-year property damage costs, derived from the Spatial Hazards Events and Losses Database for the United States,^42^ which we normalized based on state-year housing costs, measured via state-year means of the Zillow Home Value Index.^43^

### Covariates

We included yearly COVID-19 deaths per CDC reporting^44^ as a time-varying covariate. These data were used to account for changes in state-level pandemic severity, which may simultaneously lead to (1) economic changes and necessitate housing supports and (2) deaths among people experiencing homelessness.^45^

### Statistical Analysis

We first calculated summary statistics for the entire study period and for the prepandemic and postpandemic periods (PIT count years 2019-2020 and 2022-2024, respectively). As a final descriptive step, we mapped state mean values and annual means of our primary outcome variables.

We then used linear regression models to measure associations between explanatory variables (measured in the preceding year) and year-over-year change in homelessness. Models included state fixed effects to control for baseline differences in homelessness change between states, year fixed effects to control for national trends in homelessness across states, and clustered SEs by state. We first estimated bivariate associations between each explanatory variable and overall change in homelessness. We then estimated multivariable associations, including all explanatory variables in a single model of overall change in homelessness, adding the time-varying control variable for state-level pandemic severity. We ran similar multivariable models for each subgroup and secondary outcome. For the primary outcome, we then used regression parameters to derive marginal estimates of year-over-year change under observed conditions and counterfactual scenarios in which statistically significant explanatory variables were set to zero.

We conducted several sensitivity analyses to check results’ sensitivity to variable construction, sample selection, and model specification. As mentioned earlier in this section, year-over-year change can produce extreme values. Thus, we top-coded extreme values of the outcome. Second, we top-coded extreme values of the climate damage explanatory variable. Third, because determinants of change in homelessness may have evolved during the COVID-19 pandemic era, we ran regression analysis restricted to PIT counts conducted from 2022 to 2024. Fourth, because fair market rents and unemployment might plausibly lie on the causal path between other explanatory variables and homelessness, we ran a version of the multivariable model excluding these potential mediators. Fifth, we ran a version of the regression excluding one state at a time to assess the influence of individual states. Finally, our models did not explicitly measure the degree to which each explanatory variable contributed to postpandemic increases in homelessness. To answer this question, we ran a difference-in-difference model interacting each explanatory variable with a term for the prepandemic period (2019-2020) compared with postpandemic period (2022-2024).

Two-tailed *P* < .05 was used to determine statistical significance. All analyses were conducted in StataMP, version 17.0 (StataCorp LLC).

## Results

### State Year-Over-Year Change in Homeless Counts and Homelessness Prevalence

**Figure 1.**
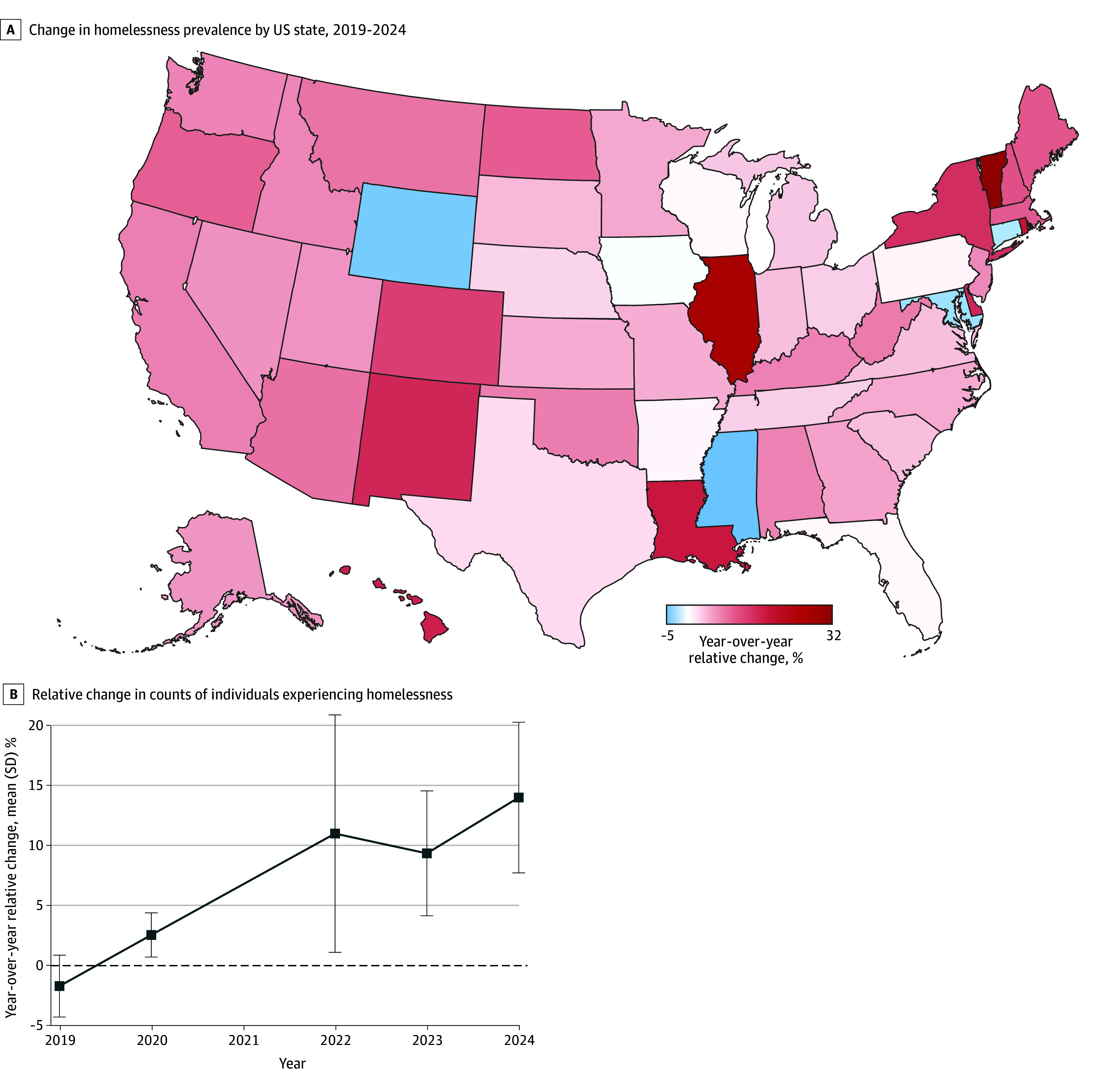
Map Showing Change in Homelessness and Line Graph Showing Yearly Change Within US States, 2019-2024 Data are shown for the prepandemic (2019-2020) and postpandemic periods (2022-2024) (N = 255 state-years). Data for 2021 were excluded because the January 2021 point-in-time count was limited due to COVID-19 pandemic restrictions.

From 2019 to 2024 (excluding 2021; N = 255 state-years), the mean (SD) year-over-year increase in homelessness was 7.0% (22.5%) (Table 1). Mean (SD) changes of 6.1% (29.1%) in sheltered homelessness, 16.0% (36.5%) in unsheltered homelessness, 7.2% (20.6%) in adult homelessness, and 8.4% (44.0%) in child homelessness were observed. The mean absolute change in overall homelessness was an increase of 1.0 (4.8) people experiencing homelessness per 10 000 population; the mean (SD) prevalence of overall homelessness was 17.4 (15.3) people per 10 000 population (Figure 1).

**Table 1.** Change in Homelessness and Explanatory Variables, 2019-2024

Variable	Mean (SD)		
	Study period (N = 255 state-years)	COVID-19 pandemic	
		Prepandemic period (n = 102 state-years)	Postpandemic period (n = 153 state-years)
**Outcome^a^**			
Year-over-year relative change in homelessness, %			
Overall (primary outcome)	7.0 (22.5)	0.4 (8.6)	11.5 (27.4)
Homelessness type			
Sheltered	6.1 (29.1)	−1.4 (8.1)	11.2 (36.2)
Unsheltered	16.0 (36.5)	11.1 (33.2)	19.2 (38.3)
Age group			
Adults	7.2 (20.6)	1.1 (8.9)	11.3 (24.8)
Children	8.4 (44.0)	−1.4 (19.1)	15.0 (53.7)
Absolute change in overall homelessness per 10 000 population, %	1.0 (4.8)	−0.1 (1.4)	1.8 (5.9)
Prevalence of overall homelessness per 10 000 population, %	17.4 (15.3)	15.6 (14.4)	18.6 (15.6)
**Explanatory^b^**			
Average rent for a 2-bedroom apartment, in $100s per mo	11.3 (3.3)	10.4 (2.9)	11.9 (3.5)
Unemployment rate, %	3.8 (1.1)	3.7 (0.8)	3.8 (1.2)
Emergency rental assistance per capita, rent-month equivalents per low-income renter household	0.3 (0.4)	NA	0.5 (0.5)
Moratorium coverage, % person-time	6.2 (20.9)	NA	10.3 (26.1)
Substance use mortality, No. of fatal overdoses per 10 000 population	27.3 (13.3)	20.4 (9.6)	31.9 (13.5)
Immigrant rate per capita, No. of immigrants per 10 000 population	50.6 (25.1)	49.1 (22.5)	51.6 (26.7)
Climate-related damages per capita, No. of home-equivalents lost per 10 000 population	1.7 (9.6)	1.3 (2.8)	2.0 (12.1)

Abbreviation: NA, not applicable.

^a^ Time points included the study period (2019-2024), prepandemic period (2019-2020), and postpandemic period (2022-2024). Data for 2021 were excluded because the January 2021 point-in-time count was limited due to COVID-19 pandemic restrictions.

^b^ Time points included the study period (2018-2023), prepandemic period (2018-2019), and postpandemic period (2021-2023).

### Explanatory Variables

Across the entire study period (2018-2023), the mean (SD) rent was $1130 ($330) per month. The mean (SD) unemployment rate was 3.8% (1.1%). During the postpandemic years (2021-2023), the mean (SD) ERA distribution was 0.5 (0.5) rent-months per capita, and eviction moratoria covered a mean (SD) 10.3% (26.1%) of person-time. The mean (SD) prevalence of overdose mortality was 27.3 (13.3) overdose deaths per 10 000 population across the study period. The mean (SD) immigration rate was 50.6 (25.1) immigrants per 10 000 population, and a mean (SD) 1.7 (9.6) home-equivalents were lost to climate-related disasters per 10 000 population. Maps and trend plots of explanatory variables are provided in eFigure 1 in Supplement 1.

### Factors Associated With Year-Over-Year Change in Homelessness

In bivariate regression analysis, moratorium coverage, immigration rate, and climate-related damages were each associated with overall changes in homelessness (Table 2). No association was observed for the remaining factors. In multivariable regression analysis, only eviction moratorium coverage and climate-related damages remained associated with changes in homelessness within states; immigration was no longer associated. Each additional 1.0% of person-time covered by a moratorium was associated with a 0.36–percentage point (pp) lower change in overall homelessness (95% CI, 0.10-0.61 pp; *P* = .01). Each home-equivalent lost to climate-related events per 10 000 population was associated with a 1.00-pp greater change in overall homelessness (95% CI, 0.78-1.22 pp; *P* = .001).

**Table 2.** Associations Between Explanatory Variables and Annual Year-Over-Year Relative Change in Homelessness in US States, 2018-2024^a^

Explanatory variable	Overall homelessness				By homelessness type				By age			
	Bivariate		Multivariable		Sheltered (multivariable)		Unsheltered (multivariable)		Adult (multivariable)		Child (multivariable)	
	Coefficient (95% CI)	*P* value	Coefficient (95% CI)	*P* value	Coefficient (95% CI)	*P* value	Coefficient (95% CI)	*P* value	Coefficient (95% CI)	*P* value	Coefficient (95% CI)	*P* value
Average rents	2.59 (−1.48 to 6.66)	.21	1.95 (−2.83 to 6.73)	.42	3.90 (−0.56 to 8.36)	.08	−1.76 (−7.32 to 3.81)	.53	1.80 (−2.79 to 6.38)	.44	2.89 (−5.19 to 10.98)	.48
Unemployment	−0.07 (−5.80 to 5.67)	.98	2.30 (−4.82 to 9.42)	.52	3.40 (−5.33 to 12.12)	.44	−1.63 (−12.40 to 9.15)	.76	1.16 (−5.71 to 8.02)	.74	5.55 (−4.24 to 15.35)	.26
Emergency rental assistance^b^	−8.32 (−22.40 to 5.80)	.24	−7.78 (−20.53 to 4.97)	.23	−8.20 (−22.43 to 6.03)	.25	15.93 (−12.32 to 44.18)	.26	−6.78 (−18.06 to 4.49)	.23	−9.20 (−28.96 to 10.55)	.35
Moratorium coverage^b^	−0.26 (−0.49 to −0.02)	.03	−0.36 (−0.61 to −0.10)	.01	−0.29 (−0.57 to −0.00)	.048	−0.40 (−0.77 to −0.04)	.03	−0.34 (−0.58 to −0.10)	.01	−0.35 (−0.70 to 0.00)	.05
Substance use mortality	0.11 (0.31-0.53)	.60	0.14 (−0.49 to 0.76)	.66	−0.18 (−0.89 to 0.53)	.61	0.74 (0.12- 1.37)	.02	0.10 (−0.47 to 0.67)	.73	0.27 (−0.69 to 1.24)	.57
Immigration rate	0.37 (0.05- 0.69)	.02	0.28 (−0.03 to 0.58)	.07	0.27 (−0.16 to 0.70)	.20	−0.09 (−0.69 to 0.50)	.75	0.26 (−0.02 to 0.53)	.06	0.34 (−0.20 to 0.87)	.22
Climate-related property loss	1.04 (0.87- 1.21)	<.001	1.00 (0.78- 1.22)	<.001	1.76 (0.96-2.56)	<.001	−0.02 (−0.15 to 0.12)	.81	0.68 (0.41- 0.95)	<.001	3.19 (2.80-3.57)	<.001

^a^ In multivariable regression analysis, coefficients were adjusted for other explanatory variables, as well as time-varying controls for state-level pandemic severity: state-year COVID-19 deaths, unemployment rate, and inflation. All models include state and year fixed effects and state-clustered SEs. Coefficients should be interpreted as percentage point changes in year-over-year change, per unit increase in each explanatory variable.

^b^ Emergency rental assistance and moratorium coverage were set to zero for the prepandemic years (2018-2019).

Regarding subgroups of homelessness, the same 2 factors (moratorium coverage and climate-related damages) were associated with year-over-year change in homelessness among adults and sheltered homelessness. Moratorium coverage and overdose mortality were associated with change in unsheltered homelessness. Only climate-related damages were associated with change in homelessness among children.

### Marginal Estimates

**Figure 2.**
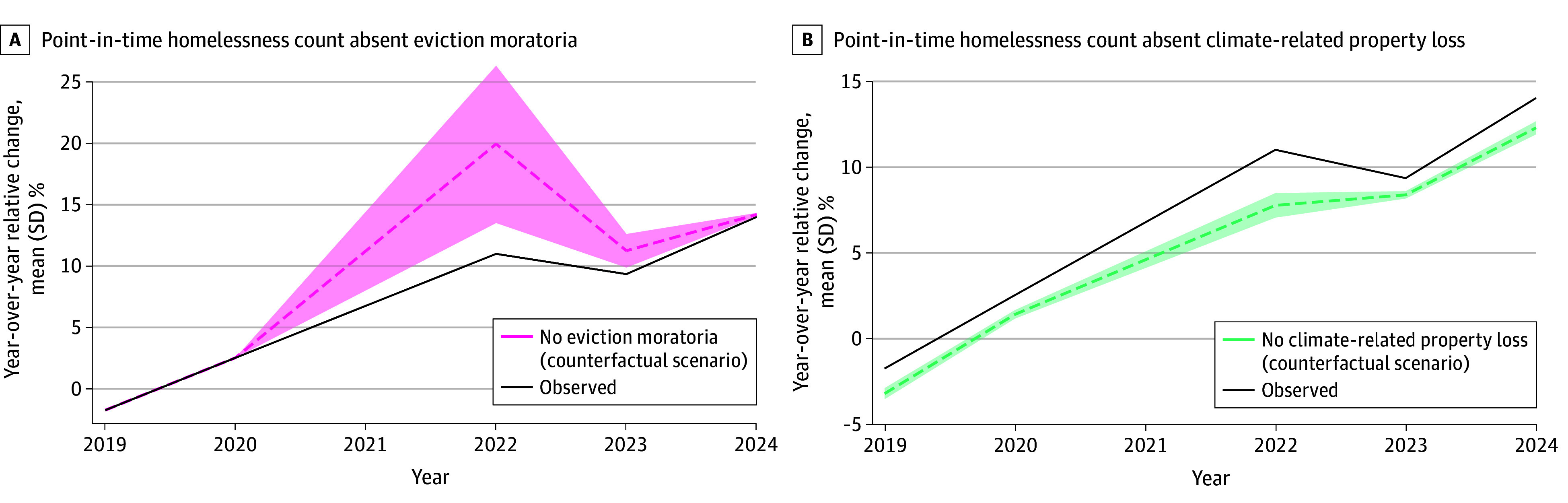
Line Graphs Showing Marginal Estimates of Annual Year-Over-Year Relative Change in Homelessness Under Observed and Counterfactual Conditions for a Typical US State, 2019-2024 Because eviction moratoria were only implemented beginning in 2020, counterfactual scenarios only apply beginning in the postpandemic period, point-in-time years 2022 to 2024.

We estimated mean change in homelessness by year, under counterfactual scenarios in which there were no eviction moratoria and, separately, no property loss due to climate-related disasters (Figure 2). Taking 2022 as an example, we observed a mean (SD) year-over-year increase in homelessness of 11.0% (36.4%) in the average state. Absent eviction moratoria, we estimated that this mean increase would have been 19.9% (95% CI, 13.5%-26.3%). Absent property loss due to climate-related disasters, the mean increase was estimated to have been 7.8% (95% CI, 7.1%-8.5%).

### Absolute Change in Homelessness Prevalence and Baseline Prevalence

When we explored absolute change in homelessness prevalence, we continued to see that eviction moratoria were associated with lower increases in homelessness (coefficient, −0.07 pp [95% CI, −0.13 to −0.02 pp]; *P* = .01) (Table 3). The association between climate-related damages and change in homelessness no longer remained (coefficient, 0.13 pp [95% CI, −0.05 to 0.31 pp]; *P* = .15).

**Table 3.** Absolute Change in Prevalence of Overall Homelessness in US States, 2018-2024^a^

Explanatory variable	Absolute change in prevalence of overall homelessness, 2018-2024 (N = 255 state-years)^b^		Baseline prevalence of overall homelessness, 2019 (n = 51 state-years)	
	Coefficient (95% CI)	*P* value	Coefficient (95% CI)	*P* value
Average rent	1.16 (−0.54 to 2.87)	.18	2.90 (1.18-4.63)	.001
Unemployment	0.00 (−1.34 to 1.35)	>.99	4.78 (1.11-8.45)	.01
Emergency rental assistance	−1.88 (−5.25 to 1.49)	.27	NA	NA
Moratorium coverage	−0.07 (−0.13 to −0.02)	.01	NA	NA
Substance use mortality	0.04 (−0.09 to 0.17)	.53	−0.29 (−0.64 to 0.05)	.09
Immigration rate	0.08 (−0.04 to 0.21)	.19	0.13 (−0.09 to 0.44)	.24
Climate-related property loss	0.13 (−0.05 to 0.31)	.15	−0.64 (−7.73 to 0.44)	.24

Abbreviation: NA, not applicable.

^a^ Unless otherwise indicated, values are reported as the change in the number of people experiencing homelessness per 10 000 population.

^b^ Absolute change*_sy_* = [Count*_y_*/10 000 population*_sy_*] – [count*_s_*_(_*_y_*_−1)_/10 000 population*_s_*_(_*_y_*_−1)_], where *s* indicates state, *y* is point-in-time year, and *y *− 1 is 1 year prior.

Analyzing the same set of explanatory variables in relationship to baseline (2019) homelessness prevalence, we found that average rents and unemployment were associated with overall prevalence of homelessness. Each additional $100 in monthly rents was associated with 2.90 additional people experiencing homelessness per 10 000 population (95% CI, 1.18-4.63 additional people per 10 000 population; *P* = .001). Each percentage point increase in the unemployment rate was associated with 4.78 additional people experiencing homelessness per 10 000 state population (95% CI, 1.11-8.46 additional people per 10 000 population; *P* = .01).

### Sensitivity Analyses

Results of sensitivity analyses (eFigure 2 and eTable 1 in Supplement 1) were very consistent with our main regression results. Difference-in-difference results (eTable 2 in Supplement 1) suggest that eviction moratoria were associated with attenuated increases in homelessness between the prepandemic and postpandemic periods. Climate-related damages, by contrast, were associated with year-over-year increases in homelessness throughout the study period but did not significantly accelerate increases after the pandemic.

## Discussion

Correctly determining the relative contributions of factors associated with rising levels of homelessness is crucial for framing the problem and developing effective solutions to prevent surges in homelessness and promote population health. Unlike prior studies, we aimed to identify factors associated with year-over-year change in homelessness, rather than absolute counts or prevalence.

In this study, we identified 2 main factors associated with state-level changes in homelessness since 2019. First, states with longer eviction moratoria during the COVID-19 pandemic had smaller year-over-year increases in homelessness, suggesting that the end of pandemic moratoria may have fueled postpandemic spikes in homelessness. Second, states that experienced more climate-related property damage experienced larger increases in homelessness. We did not find average rents, unemployment, ERA distribution, overdose deaths, or immigration to be consistently associated with recent year-over-year increases in homelessness. However, consistent with past literature,^29^ rents and unemployment appeared to be the primary factors associated with homelessness prevalence.

The COVID-19 pandemic and fears of mass eviction led to several policies designed to promote housing security. Studies have shown that eviction moratoria were associated with reduced COVID-19 spread^46,47,48,49^ and improved mental health.^50,51,52^ Our findings suggest that reductions in homelessness may be one pathway for these health outcomes. Our null results regarding ERA are somewhat consistent with published studies, which found relatively modest, protective associations with rent arrears, concern about eviction, and mental health.^53,54,55^ These somewhat muted impacts may be due to the availability of other pandemic-era financial supports^53^ or because ERA recipients were at less immediate risk for homelessness.^56^ Of note, PIT count data do not include individuals experiencing less acute forms of homelessness, such as doubling up with others, which may be more sensitive to interventions such as ERA.

In this study, climate-related property destruction was a major factor associated with relative, year-over-year change in homelessness within states. Of note, associations did not remain when we analyzed absolute change, likely because climate disasters in this period occurred in states with smaller unhoused populations (ie, places more likely to see a large relative change, but more modest absolute change). Nonetheless, this finding has notable implications, as climate-related disasters are increasing in number and intensity. Individuals in low-income households, already most at risk for homelessness, are often more likely to live in areas highly susceptible to climate change–related disasters.^57^ Even for those who do not lose their homes in disasters, property destruction has spillover effects in housing markets: increased demand for housing drives up rents for all and displaces the most vulnerable.^58,59^ Thus, housing and health effects of climate-related events extend beyond emergency periods.

Economic factors such as rents did not emerge as significant predictors of year-over-year change in homelessness. This finding has much to do with our choice of dependent variable, year-over year change: we do not expect slow-moving, structural variables such as rent to lead homelessness to spike in the short term. This is not to say, however, that economic factors are not major factors of homelessness: consistent with past analyses,^28,29^ we found that rents and unemployment were the primary factors associated with baseline homelessness prevalence within states, over the study period.

We used overdose deaths to proxy the evolving severity of substance use epidemics within states but did not find associations between this measure and homelessness change, except with respect to unsheltered homelessness. This is consistent with findings that high rates of substance use among people experiencing homelessness may be a consequence—more than a driver—of homelessness.^60^ Similarly, immigration was not associated with homelessness change, leading us to conclude that immigration is not a major factor contributing to change in homelessness, compared with the other factors we studied.

Our quasiexperimental study offers several important takeaways for US policymakers seeking to reduce homelessness and improve population health. Our analysis found null results related to immigration and substance use, suggesting that intervening on these factors alone is unlikely to prevent increases in homelessness. In contrast, we found that eviction moratoria, when implemented at scale, may substantially mitigate increases in homelessness. More broadly, this finding points to a need for population-level eviction prevention. Examples of effective policies include tenant right-to-counsel^66,67^ and eviction diversion programs.^68^ Our findings of larger percentage increases in homelessness associated with climate-related property damage underscore the notion that homelessness can be seen as a predictable consequence of climate disasters. Bearing these results in mind, the Federal Emergency Management Agency and state agencies might focus more heavily on housing stabilization in their disaster response plans, while dedicating adequate funding to provide housing-specific services. Thinking more upstream, efforts to mitigate climate change and improve the resiliency of our housing stock may reduce the risk of homelessness following disasters.

### Limitations

Our study has several limitations. First, PIT data undercount individuals experiencing homelessness.^61^ Because jurisdictions’ methodologies for conducting PIT counts remain largely consistent across years, this is unlikely to bias our findings. Second, our measures of explanatory variables are imperfect: ERA reporting has been shown to have inconsistencies^62,63^; overdose mortality reflects both substance use prevalence and drug supply lethality^64^; the American Community Survey may underestimate the count of undocumented immigrants^65^; and climate-related damages include business as well as residential losses.^42^ Each of these measurement issues ought to apply equally across states and years and thus should not strongly bias results. Third, analyses were performed at the state-year level and do not account for substate or within-year variation in either the explanatory variables or outcomes. For example, both ERA rollout and PIT counts varied tremendously within states. Fourth, there is potential for unmeasured confounding. As one example, our analysis did not account for large, state-level investments in homeless services. However, for such investments to bias results, they would have to systematically occur in the same states and in the same years as changes in other explanatory variables.

## Conclusions

In this cohort study of 50 states and Washington, DC, measured from 2019 to 2024, eviction moratoria and climate-related damages were associated with within-state, relative change in homelessness. These findings suggest that to attenuate surges in homelessness, policymakers should work to prevent evictions, address climate change, and build climate resiliency in housing.

## Notes

Supplement 1.
eMethods

eReferences
**eFigure 1.** Maps and Trend Plots of Explanatory Variables **eTable 1.** Results of Sensitivity Analyses for Year-Over-Year Change in Overall Homelessness (N = 255 State-Years, Unless Otherwise Noted) **eFigure 2.** Results of Sensitivity Analysis Excluding Single States From Regressions (n = 250 State-Years) **eTable 2.** Difference-in-Difference Sensitivity Analysis

Supplement 2.
Data Sharing Statement
