# Supplementary material for: Factors Associated With Rising Homelessness Within US States, 2019 to 2024
Source: JAMA Netw Open. 2026 Apr 6;9(4):e265187. doi: 10.1001/jamanetworkopen.2026.5187 (PMC13054623; doi:10.1001/jamanetworkopen.2026.5187)
Supplement: Supplement 1. — eMethods eReferences eFigure 1. Maps and Trend Plots of Explanatory Variables eTable 1. Results of Sensitivity Analyses for Year-Over-Year Change in Overall Homelessness (N = 255 State-Years, Unless Otherwise Noted) eFigure 2. Results of Sensitivity Analysis Excluding Single States From Regressions (n = 250 State-Years) eTable 2. Difference-in-Difference Sensitivity Analysis [file jamanetwopen-e265187-s001.pdf]

Supplementary Online Content

Leifheit KM, Robinson L, Nkansah M, Szilagyi PG, Pollack CE. Factors associated with rising homelessness within US states, 2019 to 2024. *JAMA Netw Open*. 2026;9(4):e265187. doi:10.1001/jamanetworkopen.2026.5187

eMethods

eReferences

eFigure 1. Maps and Trend Plots of Explanatory Variables

eTable 1. Results of Sensitivity Analyses for Year-Over-Year Change in Overall Homelessness (N = 255 State-Years, Unless Otherwise Noted)

eFigure 2. Results of Sensitivity Analysis Excluding Single States From Regressions (n = 250 State-Years)

eTable 2. Difference-in-Difference Sensitivity Analysis

This supplementary material has been provided by the authors to give readers additional information about their work.

© 2026 Leifheit et al. *JAMA Network Open*.

eMethods

Outcome Measures

We measured year-over-year change in state *s* for PIT year *y* as follows:

Primary outcome

$$\textit{Year-over-year change}_{sy} = (\textit{count}_{sy} - \textit{count}_{s(y-1)}) / \textit{count}_{s(y-1)}$$

Secondary outcomes

Absolutele change$_{sy}$ =

$$(\textit{count}_{y} / 10{,}000\ \textit{population}_{sy}) - (\textit{count}_{s(y-1)} / 10{,}000\ \textit{population}_{s(y-1)})$$

$$\text{Prevalence}_{sy} = [(\textit{count}_{y} / 10{,}000\ \textit{population}_{sy})$$

Regressions

We modeled homelessness change (year-over year change, absolute change) within a given state *s* and year *y* as a function of explanatory variables measured in the same state *s*, but one year prior *(y-1)* as follows:

$$Y_{sy} = Rents_{s(y-1)} + Unemployment_{s(y-1)} + ERA_{s(y-1)} + Moratorium_{s(y-1)} + SubstanceUse_{s(y-1)} + Immigration_{s(y-1)} + ClimateDamage_{s(y-1)} + State_{s} + Year_{y}$$

Prevalence regressions mirrored this, but for a single year (prevalence in 2019 regressed on explanatory variables from 2018) and omitting state and year fixed effects.

Causal assumptions: Our approach is quasi-experimental in that it uses state and year fixed effects in attempt to isolate factors contributing to change in homelessness within states.

Similar to a difference-in-difference model, these two-way fixed effects models assume parallel trends, i.e. that treated states would have trended in parallel to untreated states, had they not been treated. As a hypothetical (and extreme) example, the models assume that homelessness change in State A that blocked evictions entirely from 2021-2023 (i.e. 100% moratorium coverage; treated) would have continued on a similar trajectory to change in State B that allowed evictions to proceed (0% coverage; untreated), *had state A never implemented a moratorium.* This is a fundamentally untestable assumption. However, in this extreme case (100% treated vs. 0%, pre- and post-pandemic), we could compare pre-pandemic trends in outcomes between states A and B and, if homelessness change was trending in parallel prior to the pandemic (treatment), would conclude that the parallel trends assumption is reasonable. However, all of our explanatory variables are time-varying and continuous, meaning there is no clear delineation of treated vs. untreated or pre vs. post. In this setting, it is not possible to assess the validity of the parallel trends assumption.

Although new methods have been developed to improve upon two-way fixed effects estimators, correcting for bias related to staggered implementation[1] and interpretability in the setting of continuous treatment,[2] we are not aware of any methods which simultaneously allow for multiple continuous, time-varying treatment variables.

Bearing these limitations in mind, we are cautious in interpreting our estimated coefficients as measuring truly causal effects.

© 2026 Leifheit et al. *JAMA Network Open*.

Counterfactual estimates

We obtained counterfactual estimates by calculating linear predictions from all estimated model parameters, but replacing states' observed values for moratorium coverage and climate-related damages with zero (indicating no moratorium coverage, and no damages). Confidence intervals were calculated based on standard errors of these linear predictions.

eReferences

1. Callaway B, Goodman-Bacon A, Sant'Anna PHC. Difference-in-differences with a Continuous Treatment. *National Bureau of Economic Research*. Preprint posted online February 2024:32117. doi:10.3386/w32117

2. Callaway B, Goodman-Bacon A, Sant'Anna PHC. Difference-in-differences with a Continuous Treatment.

© 2026 Leifheit et al. *JAMA Network Open*.

eFigure 1. Maps and Trend Plots of Explanatory Variables

A. State means of explanatory variables, 2018-2023, excluding 2020 (n=255)

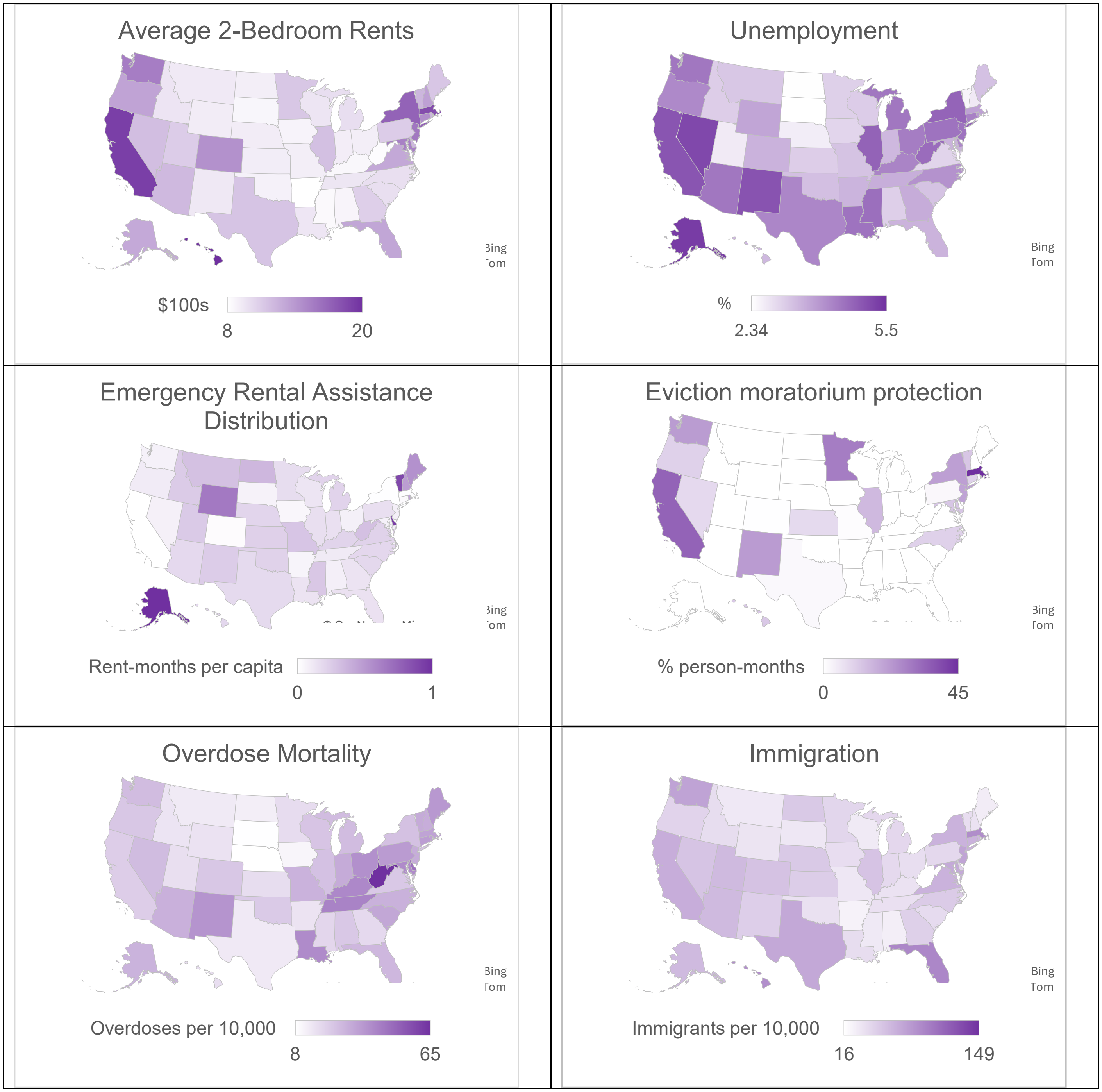

© 2026 Leifheit et al. *JAMA Network Open*.

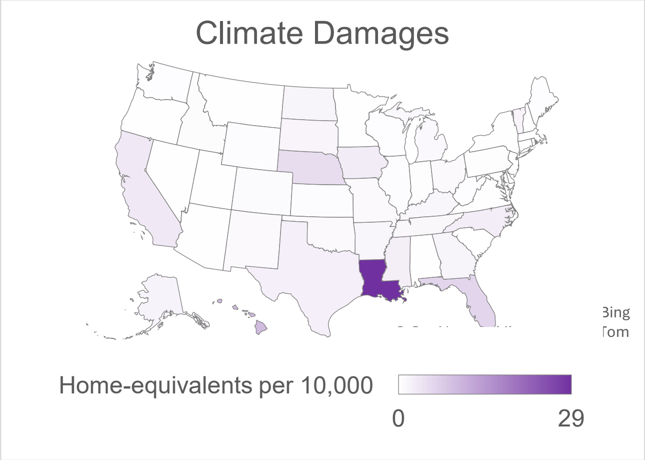

B. Plots showing within-state time trends in outcomes and explanatory variables

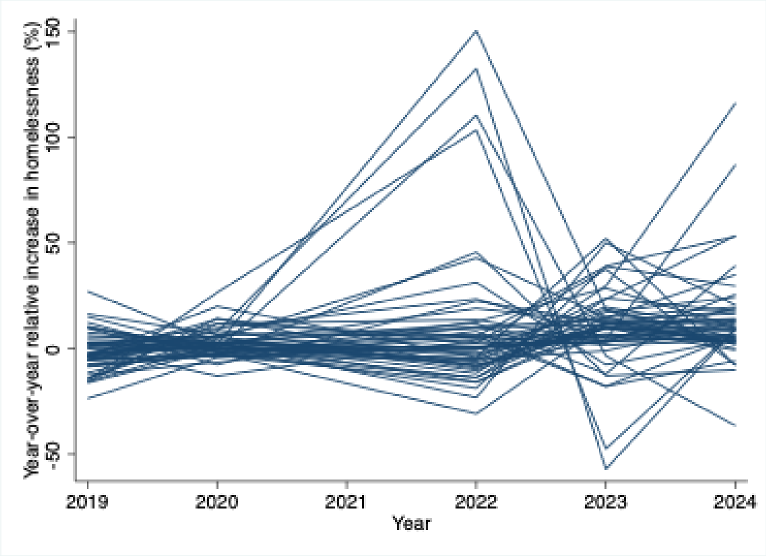

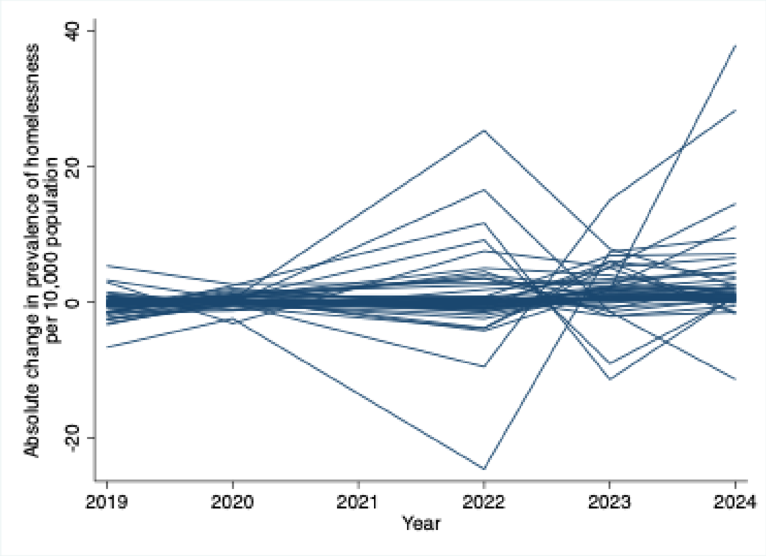

© 2026 Leifheit et al. *JAMA Network Open*.

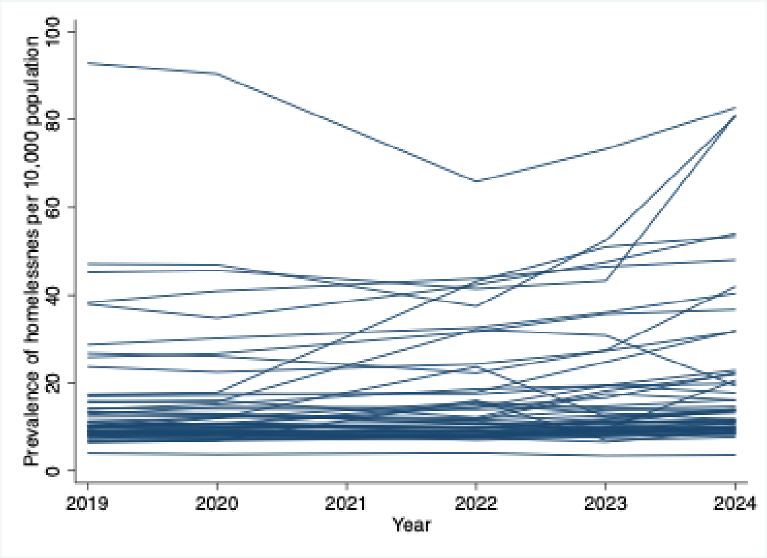

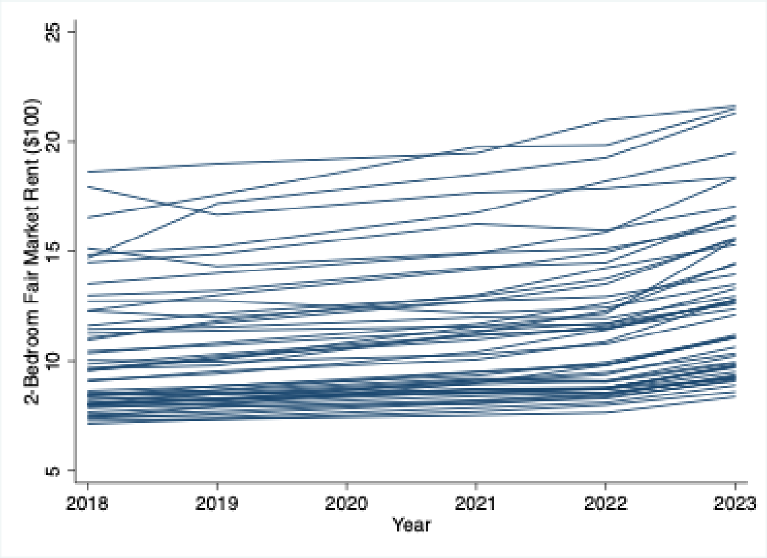

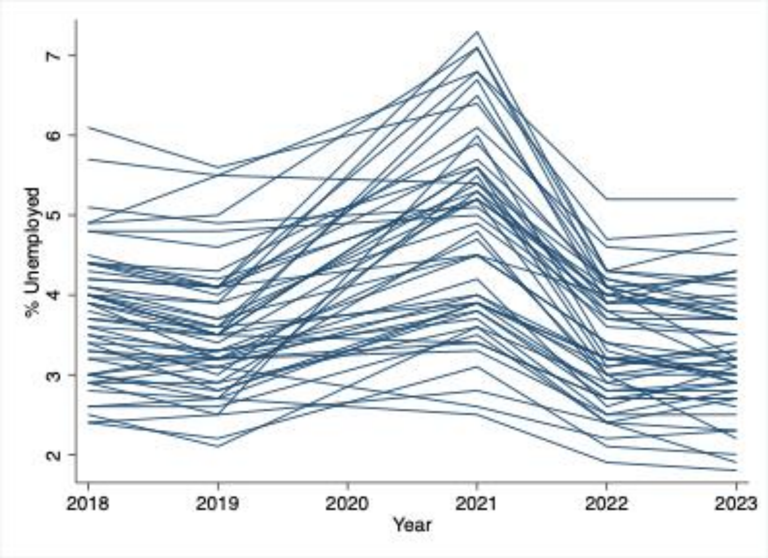

© 2026 Leifheit et al. *JAMA Network Open*.

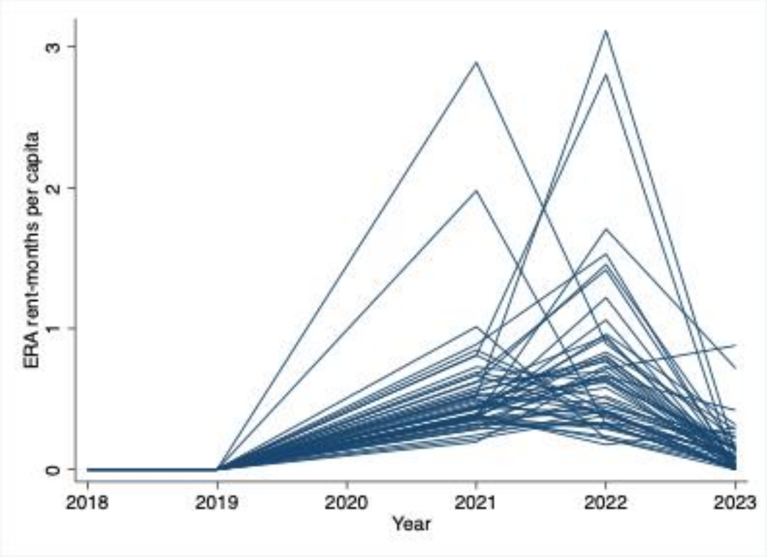

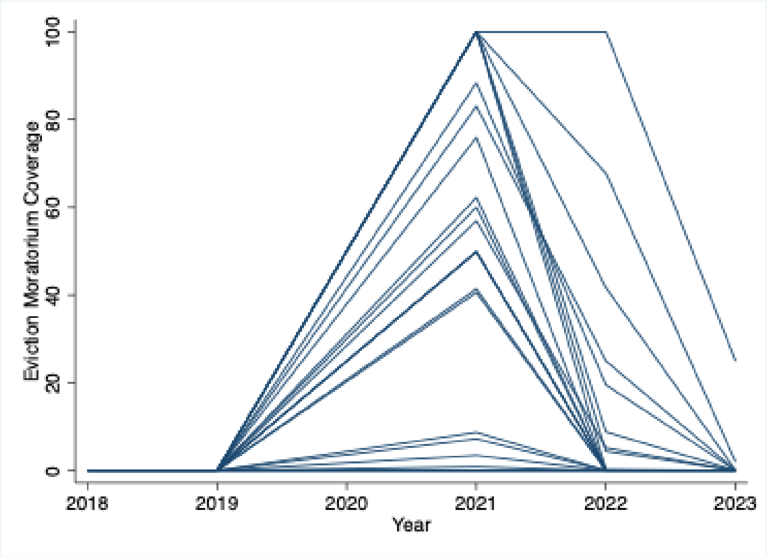

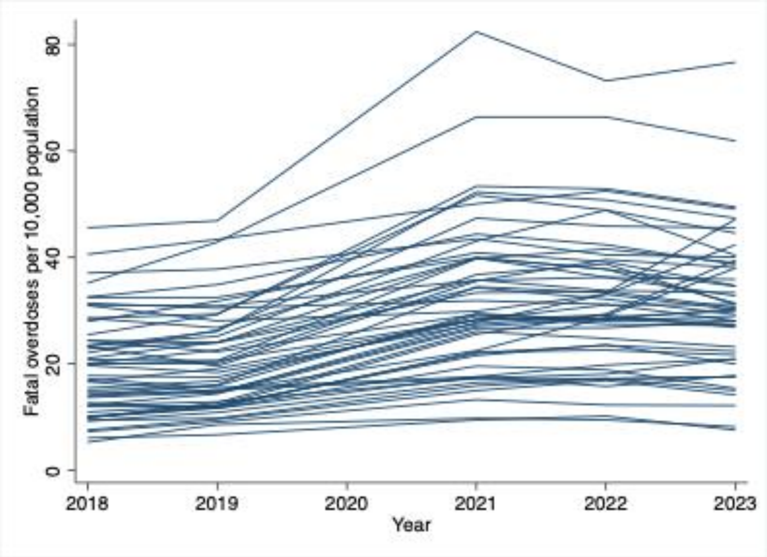

© 2026 Leifheit et al. *JAMA Network Open*.

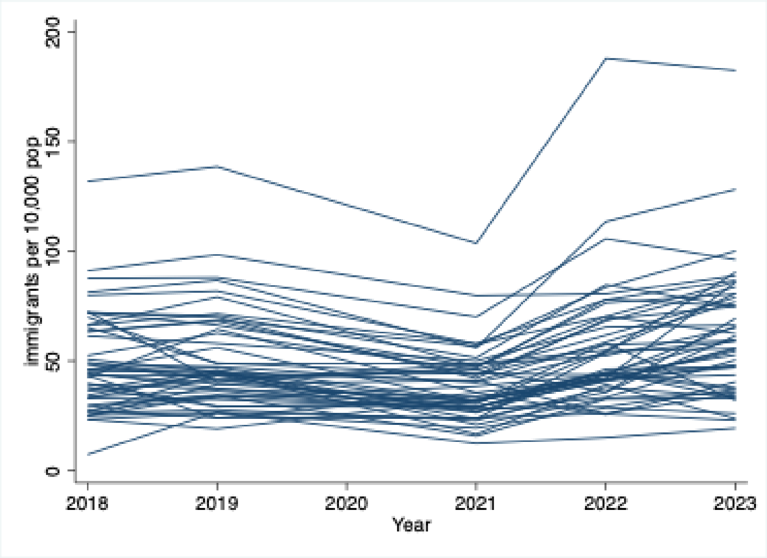

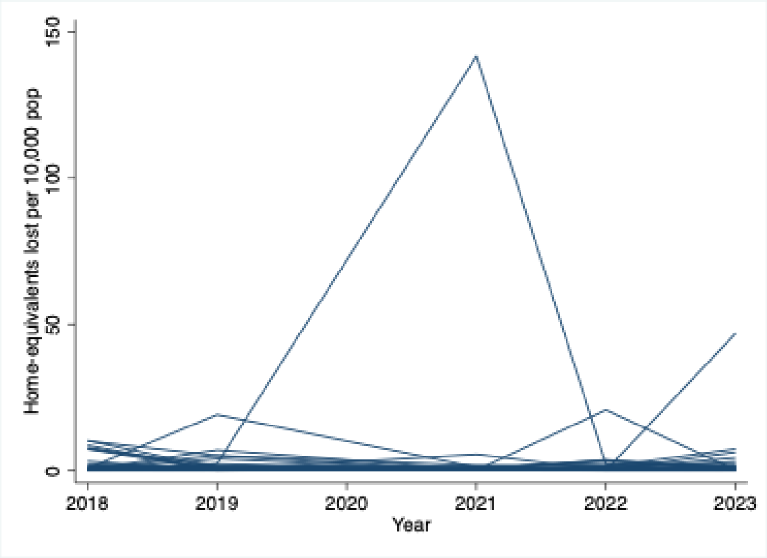

© 2026 Leifheit et al. *JAMA Network Open*.

eTable 1. Results of Sensitivity Analyses for Year-Over-Year Change in Overall Homelessness (N = 255 State-Years, Unless Otherwise Noted)

	**Main Model**		**Top-coded outcome**		**Top-coded climate damages**		**2022-2024 only (N=153)**		**Excluding rents & unemployment**	
	Coefficient (95% CI)	P-Value	Coefficient (95% CI)	P-Value	Coefficient (95% CI)	P-Value	Coefficient (95% CI)	P-Value	Coefficient (95% CI)	P-Value
Average Rents	1.95 (-2.83, 6.73)	0.417	2.46 (-1.73, 6.65)	0.244	2.55 (-2.41, 7.51)	0.307	1.16 (-10.14, 12.45)	0.838	N/A	N/A
Unemployment	2.30 (-4.82, 9.42)	0.520	0.76 (-3.74, 5.26)	0.736	2.83 (-4.51, 10.18)	0.442	2.96 (-8.94, 14.87)	0.619	N/A	N/A
Emergency Rental Assistance	-7.78 (-20.53, 4.97)	0.226	-5.53 (-17.44, 6.38)	0.356	-7.89 (-20.38, 4.59)	0.210	-10.15 (-24.67, 4.36)	0.166	-8.17 (-20.40, 4.06)	0.186
Moratorium Coverage	-0.36*** (-0.61, -0.10)	0.007	-0.28*** (-0.45, -0.10)	0.002	-0.39*** (-0.64, -0.13)	0.004	-0.47*** (-0.83, -0.12)	0.009	-0.32*** (-0.55, -0.09)	0.006
Substance use mortality	0.14 (-0.49, 0.76)	0.663	0.04 (-0.41, 0.49)	0.857	0.25 (-0.29, 0.79)	0.351	-0.86* (-1.85, 0.13)	0.087	0.11 (-0.53, 0.75)	0.727
Immigration rate	0.28* (-0.03, 0.58)	0.074	0.16* (-0.02, 0.34)	0.086	0.32** (0.00, 0.65)	0.049	0.29* (-0.01, 0.59)	0.059	0.27* (-0.02, 0.57)	0.072
Climate-related property loss	1.00*** (0.78, 1.22)	<0.001	0.44*** (0.28, 0.61)	<0.001	2.44*** (0.77, 4.10)	0.005	1.17*** (0.90, 1.44)	<0.001	1.01*** (0.80, 1.22)	<0.001

Notes:

Absolute change in rate of homeless was calculated as: *Absolute change*$_{sy}$ = *(count*$_{y}$ */10,000 population*$_{sy}$*) - (count*$_{s(y-1)}$ */10,000 population*$_{s(y-1)}$*)*

Extreme values of homelessness change = year-over-year change >50%, top-coded to 50 (N=11 state-months)

Extreme values of climate damage = damages>25 home equivalents lost per 10,000 pop, top-coded to 25 (N=4 state-months)

© 2026 Leifheit et al. *JAMA Network Open*.

eFigure 2. Results of Sensitivity Analysis Excluding Single States From Regressions (n = 250 State-Years)

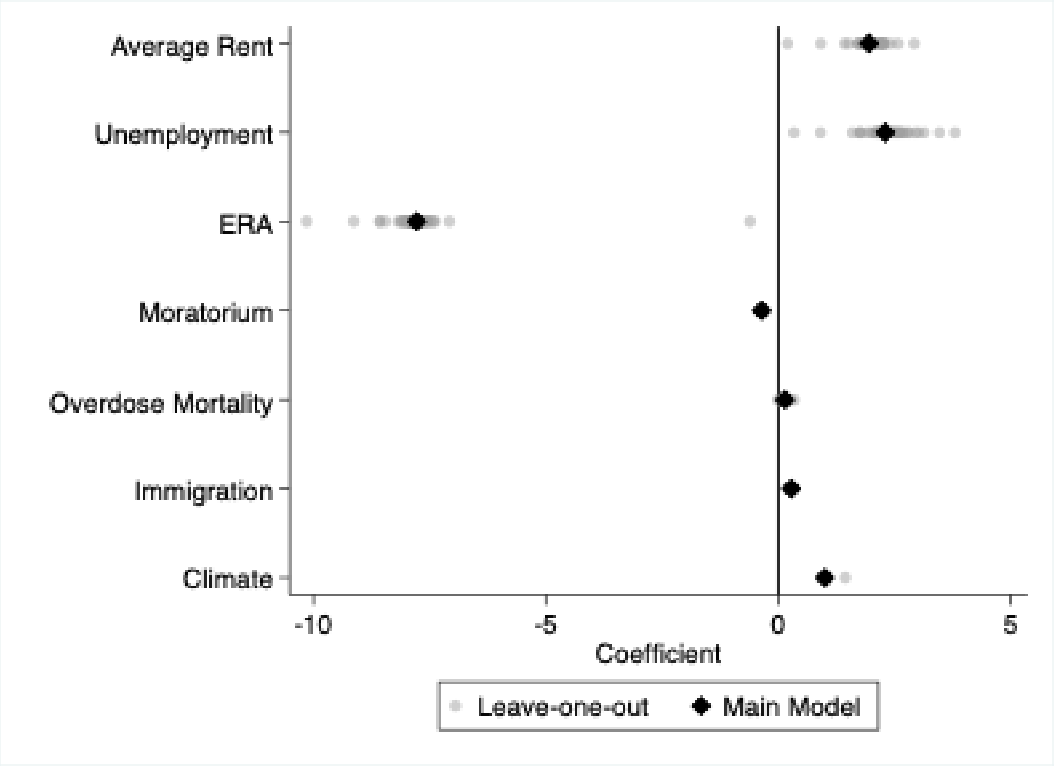

© 2026 Leifheit et al. *JAMA Network Open*.

eTable 2. Difference-in-Difference Sensitivity Analysis

	Coefficient	95% CI	p-value
COVID pre/post	4.29	-21.97, 30.55	0.744
Average Rents (FMR)			
Baseline difference	-0.08	-4.66, 4.50	0.973
DiD	1.80*	-0.02, 3.63	0.053
Unemployment			
Baseline difference	2.05	-4.07, 8.18	0.504
DiD	-1.10	-8.44, 6.24	0.765
ERA			
Baseline difference	N/A		
DiD	-8.06	-21.54, 5.43	0.236
Moratorium			
Baseline difference	N/A		
DiD	-0.36**	-0.63, -0.09	0.010
Overdose Deaths			
Baseline difference	-0.08	-0.66, 0.51	0.794
DiD	0.20	-0.23, 0.64	0.356
Immigration			
Baseline difference	0.41*	-0.05, 0.88	0.081
DiD	-0.17	-0.42, 0.08	0.182
Climate-related damages			
Baseline difference	0.60**	0.01, 1.20	0.048
DiD	0.40	-0.25, 1.06	0.222

© 2026 Leifheit et al. *JAMA Network Open*.
