# Supplementary material for: Factors Associated With Rising Homelessness Within US States, 2019 to 2024
Source: JAMA Netw Open. 2026 Apr 6;9(4):e265187. doi: 10.1001/jamanetworkopen.2026.5187 (PMC13054623; doi:10.1001/jamanetworkopen.2026.5187)
Supplement: Supplement 2. — Data Sharing Statement [file jamanetwopen-e265187-s002.pdf]

Data Sharing Statement

Leifheit. Factors Associated With Rising Homelessness Within US States, 2019 to 2024. *JAMA Netw Open*. Published April 06, 2026. doi:10.1001/jamanetworkopen.2026.5187

Data
Data available: Yes
Data types: Data (not involving human participants)
How to access data: available upon request: email kleifheit@g.ucla.edu
When available: With publication

Supporting Documents
Document types: Statistical/analytic code
How to access documents: available upon request: email kleifheit@g.ucla.edu
When available: With publication

Additional Information
Who can access the data: anyone requesting the data
Types of analyses: any purpose
Mechanisms of data availability: upon request
